# Fluid Flow Dynamics in Partially Saturated Paper

**DOI:** 10.3390/mi15020212

**Published:** 2024-01-31

**Authors:** Ashutosh Kumar, Jun Hatayama, Alex Soucy, Ethan Carpio, Nassim Rahmani, Constantine Anagnostopoulos, Mohammad Faghri

**Affiliations:** Microfluidics Laboratory, Department of Mechanical, Industrial and Systems Engineering, University of Rhode Island, 2 East Alumni Avenue, Kingston, RI 02881, USA

**Keywords:** fluid dynamics, saturated paper substrate, numerical modeling, capillary flow, porosity, pore size, HSMAC method, HORNET scheme, porous media fluid transport, Microfluid Paper-Based Analytical Devices (µPADs)

## Abstract

This study presents an integrated approach to understanding fluid dynamics in Microfluidic Paper-Based Analytical Devices (µPADs), combining empirical investigations with advanced numerical modeling. Paper-based devices are recognized for their low cost, portability, and simplicity and are increasingly applied in health, environmental monitoring, and food quality analysis. However, challenges such as lack of flow control and the need for advanced detection methods have limited their widespread adoption. To address these challenges, our study introduces a novel numerical model that incorporates factors such as pore size, fiber orientation, and porosity, thus providing a comprehensive understanding of fluid dynamics across various saturation levels of paper. Empirical results focused on observing the wetted length in saturated paper substrates. The numerical model, integrating the Highly Simplified Marker and Cell (HSMAC) method and the High Order accuracy scheme Reducing Numerical Error Terms (HORNET) scheme, successfully predicts fluid flow in scenarios challenging for empirical observation, especially at high saturation levels. The model effectively mimicked the Lucas–Washburn relation for dry paper and demonstrated the increasing time requirement for fluid movement with rising saturation levels. It also accurately predicted faster fluid flow in Whatman Grade 4 filter paper compared with Grade 41 due to its larger pore size and forecasted an increased flow rate in the machine direction fiber orientation of Whatman Grade 4. These findings have significant implications for the design and application of µPADs, emphasizing the need for precise control of fluid flow and the consideration of substrate microstructural properties. The study’s combination of empirical data and advanced numerical modeling marks a considerable advancement in paper-based microfluidics, offering robust frameworks for future development and optimization of paper-based assays.

## 1. Introduction

The exploration of fluid dynamics in Microfluidic Paper-Based Analytical Devices (µPADs) is pivotal due to their increasing application in diverse fields, such as health, environmental monitoring, and food quality analysis. Paper-based devices are characterized by their low cost, portability, and simplicity and offer significant advantages over traditional diagnostic platforms. However, a deep understanding of fluid flow within these devices is crucial to enhance their performance and broaden their application spectrum [1,2,3]. µPADs have emerged as innovative platforms for fluid handling and analysis, with their ease of fabrication and independence from complex equipment making them attractive for a wide range of applications. However, challenges such as the lack of flow control and the need for more sensitive detection methods are barriers to their widespread adoption [4,5,6]. Recent µPAD advancements showcase significant innovations in cost-effective bio-analyte analysis and diagnostics, indicating a promising future for wearable and integrated platforms [7,8].

Various theoretical models have been developed to characterize fluid dynamics in paper-based assays. These include the Lucas–Washburn (LW) equation for porous materials and models considering the porous medium as a network of capillaries. Recent research has applied the Richards equation in conjunction with two-phase flow material properties derived from image-based pore-network modeling of the filter paper. This approach addresses the shortcomings of the single-phase Darcy model, which significantly overestimates the temporal wetting penetration depths [9]. Despite these advancements, current models often fail to predict fluid behavior under varying wet conditions [10,11,12,13]. Existing models primarily focus on dry or fully saturated flow conditions, neglecting intermediate saturation levels. This limitation hinders the accurate prediction and control of fluid flow, especially in applications requiring precise fluid manipulation [14,15,16]. Moreover, the influence of factors such as paper structure, sample volume, and other attributes on flow dynamics is often inadequately addressed in these models [17,18,19].

To address the gaps in existing theories, our model aims to provide a comprehensive understanding of fluid dynamics across different saturation levels in paper substrates. By incorporating factors such as pore radius, fiber orientation, and porosity, we seek to develop a more accurate and versatile model. This advancement is motivated by the need for precise fluid control in µPADs, which is critical for enhancing their diagnostic capabilities and expanding their application range [20]. We place a particular emphasis on adapting the Kozeny–Carman model to understand fluid flow in saturated paper conditions. The Kozeny–Carman model, traditionally employed in the study of permeability in porous media, provides a semi-empirical framework that relates permeability to the structural properties of the medium, such as porosity and surface area. Its application to µPADs represents a novel approach considering the unique microstructural properties of paper. By modifying our previous model [21] to include the specific characteristics of paper, such as its fibrous network, porosity variations, and saturation effects, we aim to provide a more accurate description of fluid flow in the paper. This model is crucial for addressing the intermediate saturation states that are often overlooked in conventional models. By adapting the Kozeny–Carman equation to suit the flow dynamics of paper-based microfluidics devices, we anticipate bridging a significant gap in current models, thus enhancing the design and functionality of µPADs for a range of applications [12,13,17].

The complexity of fluid dynamics in paper-based assay is crucial for accurate control and prediction in microfluidic systems. The Navier–Stokes equations have been a cornerstone in our understanding of fluid behavior in intricate saturated environments. Enhancements in computational fluid dynamics (CFD) have further deepened our understanding and capabilities in modeling these systems. A noteworthy advancement is the development of a new computational scheme designed to calculate advection terms precisely. This innovative scheme evolves from the High Order accuracy scheme Reducing Numerical Error Terms (HORNET) methodology and is distinguished by its split operator approach, known as the Implicit HORNET [22]. Its adaptability to multidimensional problems enhances its utility in complex fluid dynamic studies. These developments collectively signify a significant stride forward in fluid dynamics modeling, combining the principles of Navier–Stokes equations and cutting-edge CFD techniques to meticulously understand fluid flow dynamics.

This study meticulously contrasts simulation-based methodologies against experimental techniques, specifically focusing on material selection challenges and the crucial role of fluid dynamics in µPADs’ efficiency and functionality. Advanced numerical methods, such as the Highly Simplified Marker and Cell (HSMAC) and the High Order accuracy scheme Reducing Numerical Error Terms (HORNET) schemes, are instrumental in simulating fluid dynamics within µPADs, accommodating a diverse range of paper saturations that are challenging, if not impossible, to achieve uniformly through experimental setups. These simulation approaches are uniquely capable of handling various fibrous materials by intricately considering properties such as porosity and fiber orientation, which are pivotal in predicting fluid flow behavior with high fidelity.

Experimental methods, while indispensable for validating the predictive capabilities of our models, encounter limitations primarily due to the difficulty in achieving consistent saturation levels and the challenge of precisely controlling experimental conditions that influence fluid behavior. Notably, our models adeptly incorporate fluid properties, including kinematic viscosity and density, to simulate fluid conductivity accurately. This nuanced comparative analysis highlights the symbiotic relationship between simulation and experimental approaches, advocating for their combined application to surmount the individual limitations of each method. Through this integrated approach, we aim to substantially advance µPAD design and application, ensuring both the precision of fluid control and the broadening of their applicability in real-world scenarios.

Thus, this work contributes significantly to the fundamental understanding of fluid flow in porous media and advances the development of more efficient and versatile models for a wide range of applications. Previous studies have primarily concentrated on fluid dynamics in poroelastic materials under dry conditions, including variations in flow rate in different µPAD dry regions due to target enzyme (*E. coli* K12) concentration [2], the Viscous Dissipation (ViDi) model for flow through micro-gaps in dry paper [10,11,17], water and oleic acid flow in dry Grade 1 paper [14], and flow comparisons across various dry Whatman filter paper grades and chromatography paper [18]. Further research has investigated flow dynamics in dry Whatman 41 filter paper [21], flow in dry paper with cut and wax boundaries [23], flow dynamics in dry poroelastic sheets [24], flow in dry fabric for different fluids [25], and the wicking dynamics of aqueous and non-aqueous fluids in swelling mediums [26,27]. While these studies focus on flow in dry regions, our research aims to provide a comprehensive model that also handles flow in partially saturated regions. The primary focus of our study is to explore the dynamics of fluid flow in partially saturated conditions, a critical aspect that has not been sufficiently addressed in existing research.

Our research presents a novel approach centered on understanding the flow complexities in paper at intermediate saturation levels and exploring the unique characteristics of paper, such as pore size and fiber orientation. Our goal is to develop a comprehensive model that deepens our understanding of fluid dynamics in µPADs, with a special emphasis on these intermediate states to optimize their real-world performance. We combine empirical experimentation with advanced computational modeling, including detailed studies of fluid flow in saturated paper substrates. Our methods, which incorporate the HSMAC method and the extended HORNET scheme, are designed to tackle the intricacies of fluid behavior in porous media, particularly at intermediate saturation levels, and consider unique paper substrate characteristics. This approach aims to precisely predict and control the flow in µPADs, thereby extending their diagnostic capabilities and applications.

## 2. Materials and Methods

This section details the comprehensive experimental framework employed to gather empirical data on fluid flow behavior within paper substrates. These experiments were designed and executed to provide robust and reliable data, which is essential for validating the results obtained from our numerical modeling efforts.

### 2.1. Materials and Experiment Flow

For the study of flow dynamics in saturated paper substrates, we used Whatman filter papers, Grade 41 (GE Healthcare 1441866) and Grade 4 (GE Healthcare 1004917), from Thermo Fisher Scientific (Waltham, MA, USA), and 0.010″ thick Backing Cards from DCN, USA, for assembly. Visualization was aided by food coloring from Wilton Icing Colors, USA. The experiments used ASTM Type 1 deionized water (LabChem-LC267405, Zelienople, PA, USA) with high resistivity. Samples were created using CorelDraw X6 2022 v24.1 and precisely cut in a cross-machine direction (CMD) and machine direction (MD) with an Epilog mini 40 W 800 Laser System. Fluid flow recording and analysis were conducted using an 8 megapixel video camera at 30 fps, with Avidemux 2.8.1 for playback and data collection.

**Figure 1:**
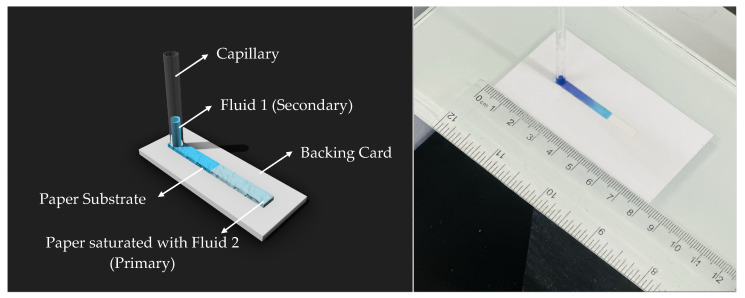
Experimental model (on the **left**) and laboratory setup (on the **right**) for the flow dynamics in the paper.

The Whatman filter paper, cut to the desired dimensions of 4 mm width using a laser cutter, was affixed to the backing card. To ensure precision and consistency during the experiments, a custom-designed fixture was used to position the sample and capillary accurately, as shown in Figure 1. This arrangement played a crucial role in reducing variability and enhancing the reliability of the results.

### 2.2. Modeling—Flow in Saturated Paper

To prepare the paper substrate, we first achieved saturation by pipetting a specific volume of fluid onto it, carefully controlling the paper’s saturation level. This was followed by a waiting period to allow the fluid to uniformly spread across the paper. Subsequently, a borosilicate capillary tube with a 1 mm radius was employed to introduce a defined amount of fluid onto the saturated paper. This setup facilitates the transfer of the sample fluid from the loading zone to the distal end through capillary action.

**Figure 2:**
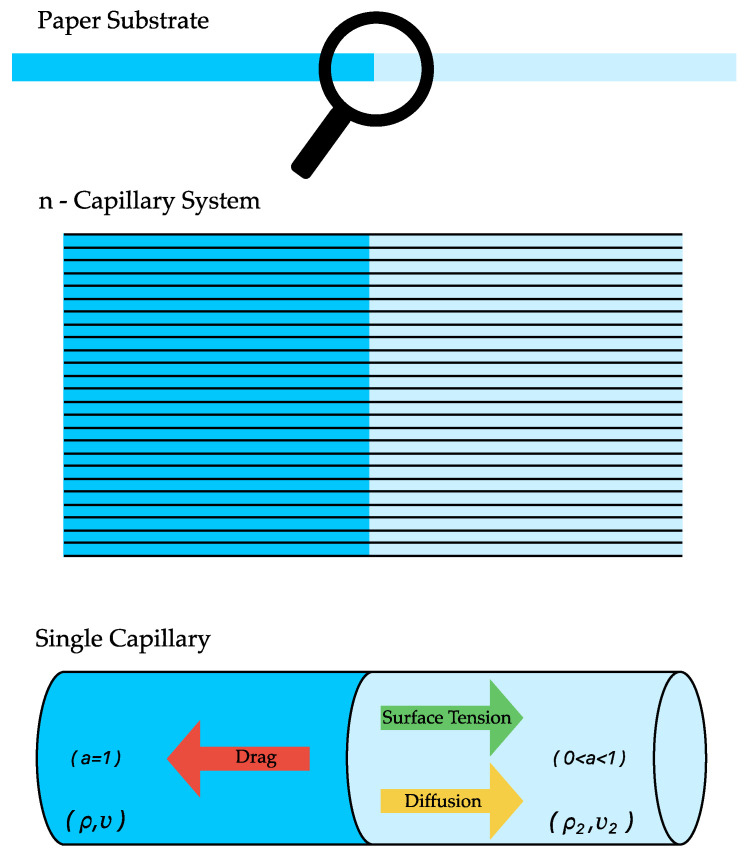
Flow dynamics in porous and saturated paper substrate.

In this section, we present our approach to modeling fluid flow within saturated paper substrates, a methodological development based on the findings of our previous study [21]. Recognizing the complexity inherent in such media, our model introduces a modified permeability factor based on the Kozeny–Carman model. This factor is specifically tailored to account for the effects of both porosity and saturation levels within the paper substrate, key variables that significantly influence fluid transport dynamics (Please see Figure 2, which depicts the flow porous and the saturated medium). This numerical model aims to provide a more nuanced and accurate representation of fluid behavior in different saturation levels of paper substrates. The method details the process of adapting traditional permeability concepts to the specific challenges presented by saturated paper mediums, ensuring a robust and applicable framework for our study.

Fluid dynamics within the paper substrate are intricately governed by the Navier–Stokes (NS) equations, which are fundamental for elucidating the momentum and continuity of fluids. Despite their complexity, these equations are essential for comprehending the subtle flow patterns in such systems. Our study focuses on modeling fluid flow within the filter paper layer, treating it as a permeable substrate influenced by both porosity and saturation level. In this manuscript, we introduce a novel modeling approach tailored for fluid flow in saturated regions. Our exploration specifically delves into the complexities of flow, employing the Volume of Fluid (VOF) model, which integrates advection and diffusion terms. This modeling technique highlights the significance of capillarity in fluid flow by incorporating the effects of surface tension and drag forces. Simplifying the continuity and momentum equations in one dimension yields the following expression:

(1) $$\frac{\partial u}{\partial t}=-\frac{1}{\rho }\frac{\partial P}{\partial x}+\left(\frac{\sigma }{\rho r}\right)\frac{\partial a}{\partial x}-u\left(\frac{v}{k^{\prime }}\right)$$

where, u is the velocity of fluid flow, $\frac{\partial P}{\partial x}$ is the pressure gradient, σ corresponds to the surface tension of fluid, ρ is the density, v is the kinematic viscosity, r is the radius of the curvature of the fluid interface in the micro-capillary, $k^{\prime }$ is the modified permeability, a is the saturation level, and the parameters $C=\frac{\sigma }{r}$ and $D^{\prime }=\frac{1}{k^{\prime }}$ [21].

As can be seen from Equation (1), the convection term, $\left[u\frac{\partial u}{\partial x}\right]$, has been omitted in accordance with the continuity equation. Furthermore, the fluid properties, such as density and kinematic viscosity, can be expressed as a function of saturation level $\left(a\right)$, as shown below:

(2) $$\rho =\left(1-a\right)\rho_{1}+a\rho_{2}$$

(3) $$v=\left(1-a\right)v_{1}+av_{2}$$

Here, indexes 1 and 2 are for primary and secondary fluids, respectively.

The saturation level, can be obtained by solving the diffusion equation bellow:

(4) $$\frac{\partial a}{\partial t}+u\frac{\partial a}{\partial x}=\psi^{\prime }\frac{\partial^{2}a}{\partial x^{2}}$$

where, $\psi^{\prime }$ is the diffusivity [20].

The adapted Kozeny–Carman model [28], as applied to flow dynamics in paper substrates, establishes a relationship between porosity (ϕ) and characteristic pore size $\left(d_{c}\right)$ and incorporates considerations for fiber orientation using the constant $60<k_{KC}<120$.

(5) $$k^{\prime }=\frac{{d_{c}}^{2}\phi^{3}}{k_{KC}{\left(1-\phi \right)}^{2}}$$

Here, the porosity in dry state is related to the saturation level of the paper substrate, as $\phi =\phi_{dry}\left(1-a\right)$. This allows for a more comprehensive understanding of fluid behavior, considering the unique structural properties of the paper substrate.

### 2.3. Numerical Method

**Figure 3:**
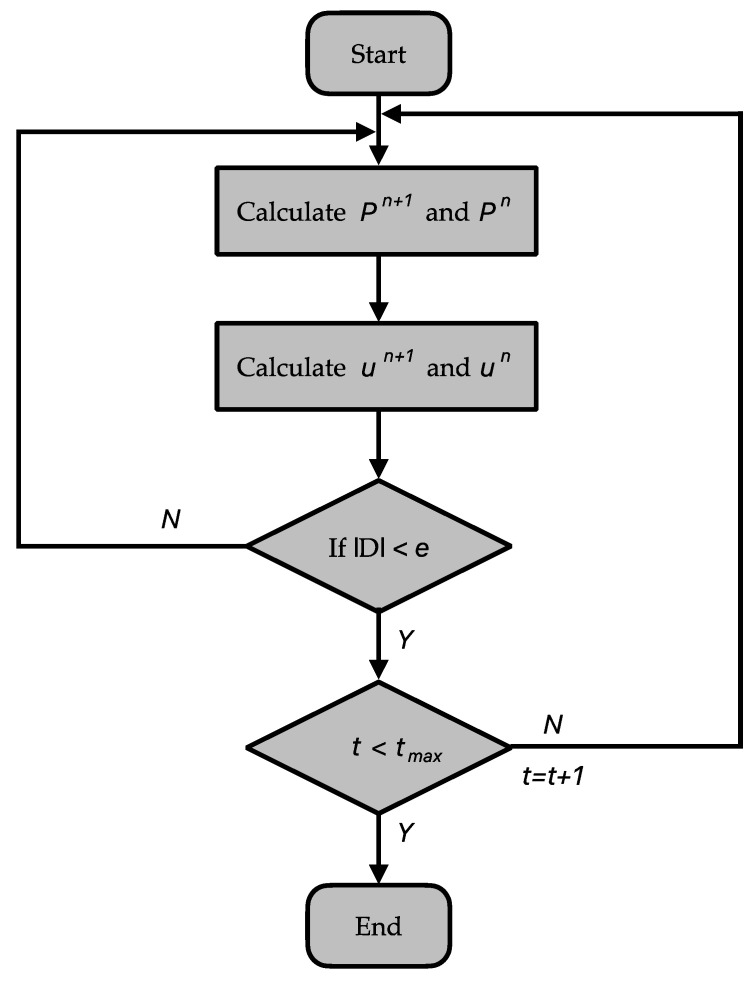
Flow chart of the HSMAC method in MATLAB.

The flow dynamics in this study are characterized by velocities tailored for capillary flow within the porous and saturated paper substrate (as per Equations (1)–(5)). These equations are solved using the HSMAC method, a technique adept at addressing unsteady fluid flow scenarios [29]. Figure 3 presents a flowchart detailing the HSMAC method executed in MATLAB. Iteratively, the method adjusts pressure (P) and velocity (u) between spatial steps (n) and (n+1), ensuring mass conservation by minimizing the divergence (D) of the velocity field. The algorithmic workflow, as illustrated, initiates the computation of pressure and velocity for the new time step (n + 1) based on the values from the previous time step (n). The model vigilantly checks for the convergence criterion: whether the divergence of the velocity field is less than a predetermined error threshold (ε). If this condition is satisfied, it implies that the flow solution has achieved the desired stability and accuracy. Should the divergence not meet the specified threshold, the model persists in its iterations until either the convergence criterion is met, or a set maximum number of iterations is reached.

This iterative refinement is pivotal for maintaining a divergence-free velocity field, a crucial characteristic for the faithful simulation of fluid flow within µPADs. The algorithm’s loop continues to execute, refining the pressure and velocity calculations, until convergence is assured by the threshold criterion, or the maximum iteration limit is encountered. It is this rigorous approach that ensures that the model’s outputs are not only numerically stable but also physically representative of the actual fluid dynamics taking place within the microfluidic environment of the µPADs.

**Figure 4:**
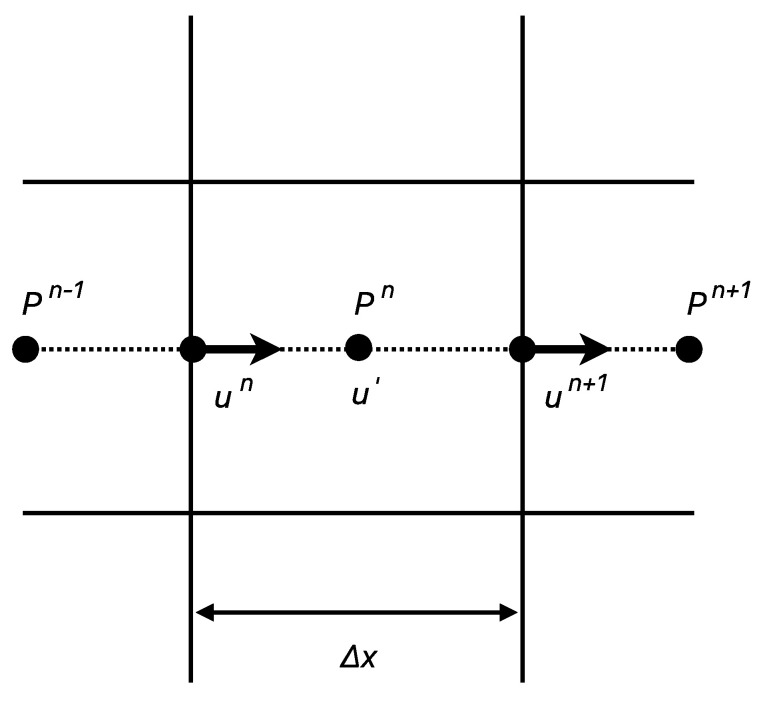
Staggered grid for flow velocity and pressure.

Using this approach, we captured the velocity profile of fluid flow within the paper substrate. By tailoring the solution to account for the specific porosity and saturation level of the paper substrate as described by the Kozeny–Carman model, we further refined our analysis. This method uses a staggered grid, as presented in Figure 4, to simulate incompressible fluid flows. The staggered grid approach is designed to prevent the numerical oscillations commonly associated with collocated grids, ensuring that the pressure and velocity fields are computed in an offset manner for enhanced accuracy. Calculating pressure at the grid’s center and velocity at its edges for improved numerical stability, the flow and pressure fields are updated using a Taylor expansion, allowing for precise estimations of these parameters as the fluid system evolves [21]. Building upon our previous work, the intermediate velocity defined at the center of the grid was calculated using the following equation:

(6) $$u^{\prime }=u^{n}+\Delta t\left(-\frac{GP}{\rho }+F\right)$$

where F and G denote the force and gradient, respectively; the net force component $\left[\left(\frac{\sigma }{\rho r}\right)\frac{\partial a}{\partial x}\right]$ represents the surface tension as per the Continuum Surface Force (CSF) model [30]; and $\left[u\left(\frac{v}{k^{\prime }}\right)\right]$ corresponds to the drag term [27], as per Darcy’s Law, with modified permeability [28].

Having determined the flow velocity using the HSMAC method, the next step focused on the impact of moisture diffusion, which is vital for the precise modeling of fluid migration within the saturated paper substrate. This task was accomplished using the HORNET scheme, which was specifically devised to address advection–diffusion in the VOF model. The key challenge lies in effectively balancing the advection $\left[u\frac{\partial a}{\partial x}\right]$ and diffusion $\left[\psi^{\prime }\frac{\partial^{2}a}{\partial x^{2}}\right]$ terms to simulate the system’s physical dynamics accurately. The HORNET scheme, thus, provides the method for resolving the one-dimensional advection-diffusion relation, detailed in Equation (4), as follows [22]:

(7) $$a_{i}^{n+1}=\left(\;\beta +K\;\right){\;a}_{i+1}^{n}+\left(\;1-\alpha \;\theta -2\;\beta -2\;K\;\right){\;a}_{i}^{n}+\left(\;\alpha \;\theta +\beta +K\;\right){\;a}_{i-1}^{n}-\alpha \;\left(\;1-\theta \;\right){\;a}_{i}^{n-1}+\alpha \;\left(\;1-\theta \;\right){\;a}_{i-1}^{n-1}$$

(8) $$K=-\frac{1}{2}\left[\alpha -\alpha^{2}\left(3-2\theta \right)\right]$$

(9) $$\theta =\frac{2\alpha^{2}-3\alpha +1-12\beta }{3\left(\alpha^{2}-\alpha -2\beta \right)}$$

where, $\left[\alpha =\left(\frac{u\Delta t}{\Delta x}\right)\right]$ is the Courant Number, $\left[\beta =\left(\frac{\psi^{\prime }\Delta t}{{\Delta x}^{2}}\right)\right]$ is the diffusion number, and Δt and Δt are the computational grid interval for space and time, respectively.

**Figure 5:**
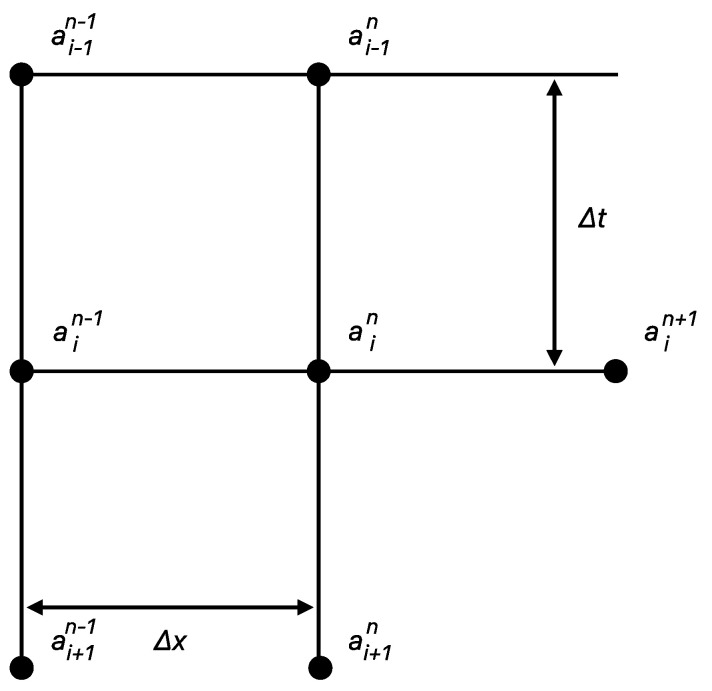
Grid for moisture migration in the paper substrate.

The HORNET scheme achieves high-precision results. However, this scheme cannot deal with negative Courant Numbers. Therefore, an extended HORNET scheme stipulating that α<0.5 was proposed. Consequently, Equation (4) was discretized in the implicit form using the grid shown in Figure 5. The grid represents the discretized domain used for modeling moisture migration within the paper substrate. The grid cells are strategically arranged to optimize the resolution of moisture diffusion and advection processes, which is crucial for accurately simulating the wicking behaviors inherent in μPADs. This methodical arrangement allows for a fine-tuned analysis of the moisture transport, providing a more detailed and accurate depiction of fluid behavior within the porous medium.

(10) $$\frac{a_{i}^{n+1}-a_{i}^{n}}{∆t}+u\left[\theta \frac{a_{i}^{n}-a_{i-1}^{n}}{∆x}+\left(1-\theta \right)\frac{a_{i}^{n-1}-a_{i-1}^{n-1}}{∆x}\right]=\psi^{\prime }\frac{a_{i+1}^{n}-2a_{i}^{n}+a_{i-1}^{n}}{{∆x}^{2}}$$

This represents the general form of the Crank–Nicolson method when applied with numerical diffusion. To ensure stability, the Courant Number was limited to less than 0.5. Thus, Equation (10) was modified as follows:

(11) $$\frac{a_{i}^{n+1}-a_{i}^{n}}{∆t}+\frac{u+\left|u\right|}{2}\left[\theta \frac{a_{i}^{n}-a_{i-1}^{n}}{∆x}+\left(1-\theta \right)\frac{a_{i}^{n-1}-a_{i-1}^{n-1}}{∆x}\right]+\frac{u-\left|u\right|}{2}\left[\theta \frac{a_{i+1}^{n}-a_{i}^{n}}{∆x}+\left(1-\theta \right)\frac{a_{i+1}^{n-1}-a_{i}^{n-1}}{∆x}\right]=\psi^{\prime }\frac{a_{i+1}^{n}-2a_{i}^{n}+a_{i-1}^{n}}{{∆x}^{2}}$$

Isolating $a_{i}^{n+1}$, we get,

(12) $$a_{i}^{n+1}=p_{1}{\;a}_{i+1}^{n}+p_{2}{\;a}_{i}^{n}+p_{3}{\;a}_{i-1}^{n}+p_{4}{\;a}_{i+1}^{n-1}+p_{5}{\;a}_{i}^{n-1}+p_{6}{\;a}_{i-1}^{n-1}$$

(13) $$K_{e}=-\frac{1}{2}\left[\left|\alpha \right|-\alpha^{2}\left(3-2\theta \right)\right]$$

(14) $$\theta_{e}=\frac{2\alpha^{2}-3\left|\alpha \right|+1-12\beta }{3\left(\alpha^{2}-\left|\alpha \right|-2\beta \right)}$$

(15) $$p_{1}=\left(\;\beta +K_{e}-\frac{\alpha -\left|\alpha \right|}{2}\theta_{e}\right)$$

(16) $$p_{2}=\left(\;1-\left|\alpha \right|\;\theta_{e}-2\;\beta -2\;K_{e}\;\right)$$

(17) $$p_{3}=\left(\;\beta +K_{e}+\frac{\alpha +\left|\alpha \right|}{2}\theta_{e}\right)$$

(18) $$p_{4}=-\frac{\alpha -\left|\alpha \right|}{2}\left(\;1-\theta_{e}\;\right)$$

(19) $$p_{5}=-\left|\alpha \right|\;\left(\;1-\theta_{e}\;\right)$$

(20) $$p_{6}=\frac{\alpha +\left|\alpha \right|}{2}\left(\;1-\theta_{e}\;\right)$$

Here, Equations (15)–(20) represent the parameters used in Equation (12), employing modified coefficients as presented in Equations (13) and (14).

In the extended HORNET scheme, the critical coefficients α and β were established, which are instrumental in capturing the advection and diffusion effects, thus ensuring the accuracy of the solution. Additionally, the updated coefficients $K_{e}$ and $\theta_{e}$ are employed to enhance the scheme’s stability, particularly in scenarios with high advection, to maintain a balance between precision and computational robustness. This method effectively tracks the temporal progression of moisture diffusion within the saturated substrate.

## 3. Results

This study presents the findings from both experimental investigations and a numerical model designed to examine fluid flow within a saturated paper substrate. Additional factors arising from the model are considered and discussed. The results detailed herein were derived using the parameters listed in Table 1, ensuring a comprehensive understanding of the system’s behavior under study.

### 3.1. Empirical Fluid Flow

The empirical results of our study primarily focused on observing the wetted length of the saturated paper substrate and the corresponding time. The full saturation level, designated as a=1, was defined by the requirement to achieve 100% saturation of the paper substrate, as determined in our previous study [20].

**Figure 6:**
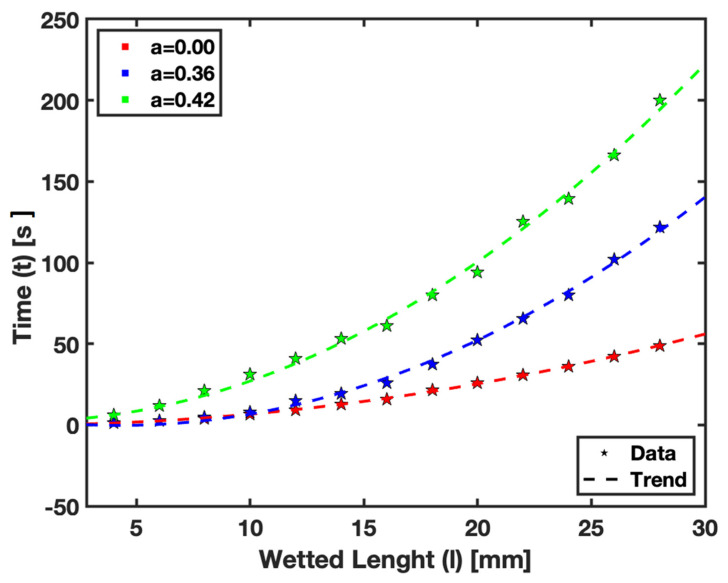
Experimental Fluid Flow in Saturated Paper Substrates.

In Figure 6, we plot the time against the wetted length for different saturation states: a=0.00  indicating dry paper and a=0.36 and a=0.42 corresponding to 10 µL and 12 µL of fluid used for saturating the paper, respectively. These measurements were taken to record the wetted length at the respective saturation levels of the paper. It is important to note that the wetted length was recorded at every 2 mm interval to ensure detailed data capture.

An additional case was investigated with a=0.50, which corresponds to 14 µL of fluid. However, the results from this saturation level were not included in the final analysis because the excessive saturation caused the fluid to flow over the surface of the paper rather than being absorbed into it. This phenomenon, attributed to limitations in our experimental setup, deviated significantly from the expected capillary action in the paper substrate. As a result, we determined the saturation level to be too high for meaningful analysis under our current experimental conditions. The experimental results of the study underscore the critical nature of controlling saturation levels in µPADs to ensure accurate and reliable fluid flow behavior, which is essential for the practical application of these devices in various fields.

### 3.2. Numerical Fluid Flow

**Figure 7:**
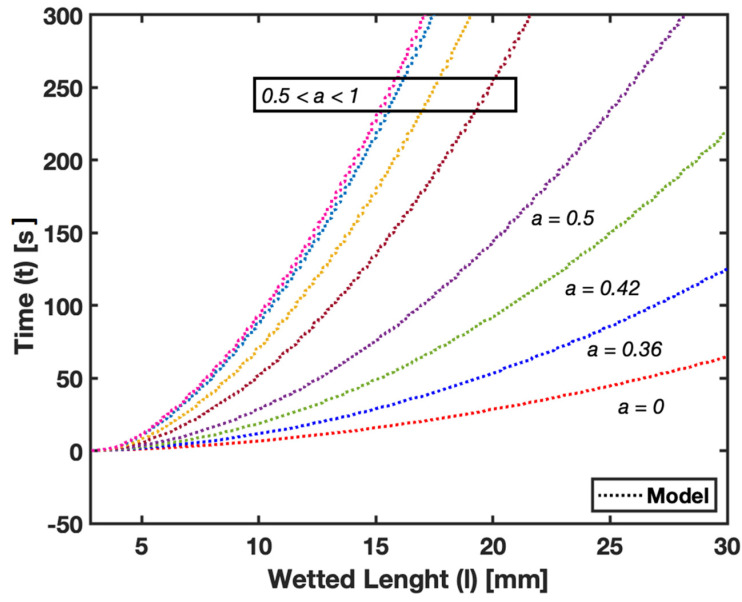
Numerical Fluid Flow in Saturated Paper Substrates.

The numerical results of our study, depicted in Figure 7, illustrate the wetted length of saturated paper against the corresponding time. These results were obtained using the combined HSMAC method and the HORNET scheme, as detailed in the numerical method section of this study. The study used various saturation levels, including a=0, 0.36, and 0.42, as well as several higher levels within the range 0.5<a<1. The fluid flow at the saturation levels above a=0.5 could not be experimentally obtained due to limitations in our experimental setup. However, our numerical model could predict the flow profile at these higher saturation levels.

The numerical analysis was particularly crucial for understanding fluid behavior at high saturation levels, where experimental observation is challenging. For instance, at the saturation level a approaching 1, where 1 represents 100% saturation, practical challenges arise. In such a state, a fully saturated paper either will not absorb additional fluid or the fluid movement becomes extremely slow. This is demonstrated in our model by an asymptote indicating that reaching a wetted length at this saturation level would take an impractically long time.

These findings highlight the utility of numerical models in predicting fluid dynamics in scenarios where experimental limitations hinder data collection. The results from the numerical simulations are essential for providing a comprehensive understanding of fluid behavior across the entire spectrum of saturation levels in µPADs. This understanding is crucial for optimizing the design and functionality in practical applications where precise fluid control is required.

### 3.3. Parametric Study

To demonstrate the adaptability of our numerical model, we present a parametric study that showcases the model’s capability to handle variables such as pore size and fiber orientation in filter paper.

**Figure 8:**
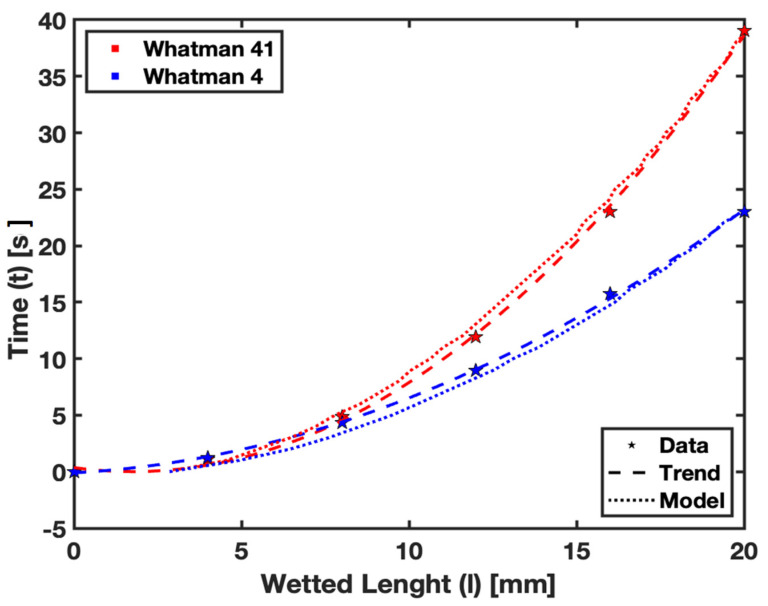
Pore Size—Flow in Whatman Grade 41 vs. Grade 4 filter papers.

In Figure 8, we examine the impact of pore size on fluid flow in Whatman Grade 41 and Whatman Grade 4 filter papers. This analysis is facilitated by using the characteristic pore size ${(d}_{c})$ parameter. Although Whatman 41 and Whatman 4 filter paper exhibit similar material properties, the latter has an effective pore size of approximately 25 µm, which results in faster fluid flow compared with Whatman 41, with an effective pore size of around 20 µm. The model accurately predicts this difference, as illustrated in the plots, demonstrating faster flow in Whatman 4 due to its larger pore size. Empirical data supporting this observation can be found in Table 2, which provides details on the wetted length upon fluid imbibition in these filter papers.

**Figure 9:**
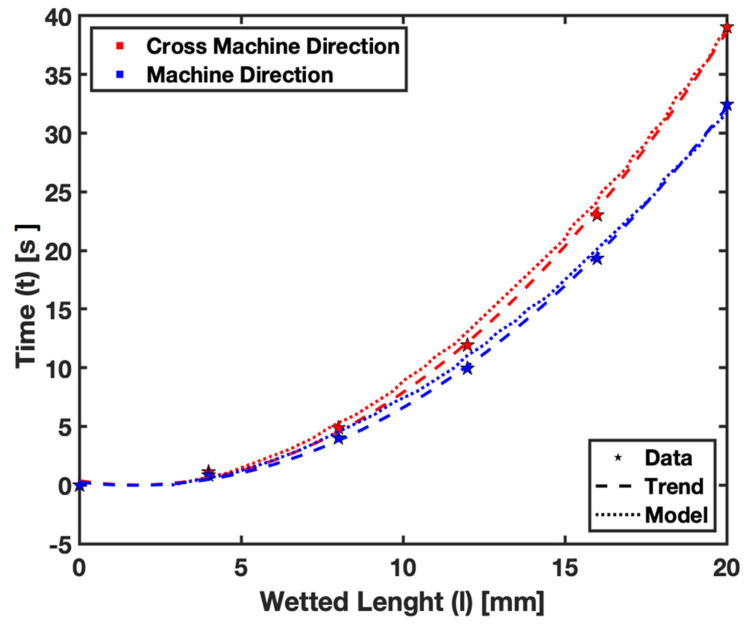
Fiber Orientation—Flow in Whatman Grade 41 filter papers.

Additionally, Figure 9 focuses on the effect of fiber orientation. Our previous study [18] showed that the flow rate significantly differs between cross-machine and machine directions. The model accounts for fiber orientation through the $k_{KC}$ constant. By adapting $k_{KC}\sim 120$ for cross-machine direction and $k_{KC}\sim 90$ for machine direction, the model can predict the flow variation due to different fiber orientations. The plot compares the flow in Whatman Grade 41 filter paper using both experimental data and the numerically predicted trend. The results show that the model can accurately predict the flow considering the fiber orientation. For detailed empirical results supporting this aspect, refer to Table 3, which provides a comprehensive dataset of the wetted length upon fluid imbibition in both machine and cross-machine fiber orientations.

These parametric plots are crucial for illustrating the versatility and applicability of our numerical model. They demonstrate the model’s ability to not only accommodate but also accurately predict changes in fluid dynamics resulting from variations in pore size and fiber orientation. This adaptability is essential for designing paper-based assays that are efficient and reliable across a range of different substrates and conditions. The model’s efficacy in predicting these variations enhances its utility in the development and optimization of µPADs for diverse applications.

### 3.4. Limiting Case

**Figure 10:**
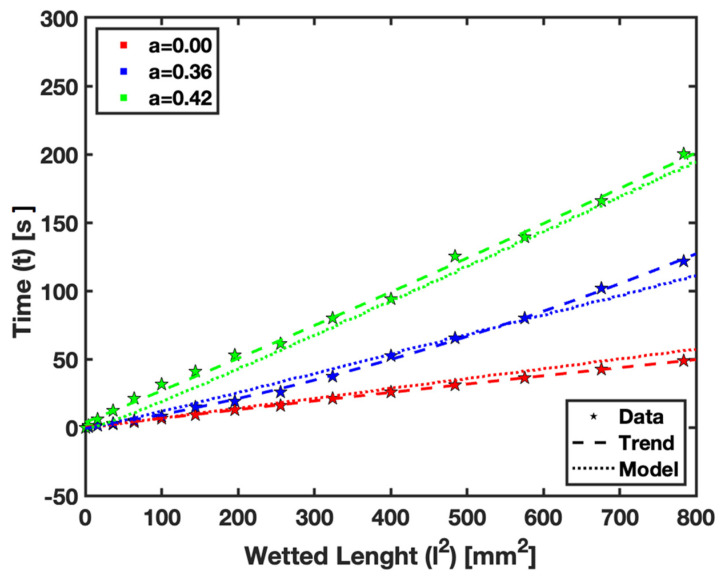
Comparison for empirical data and numerical prediction for fluid flow in saturated paper.

In this section, we focus on comparing the empirical and numerical modeling results for fluid flow in saturated paper substrates at the limiting cases of the saturation levels a=0.00, 0.36, and 0.42. This comparison serves to validate the numerical model. Figure 10 presents plots of the square of the wetted length against time for fluid flow, and the results are subsequently analyzed.

The plots demonstrate that the numerical model can accurately predict the observed behavior of fluid flow in saturated paper for the given saturation levels. Specifically, the case of a=0 represents the Lucas–Washburn relationship in dry paper. As the saturation level increases, the slope of the linear relationship also increases, indicating that more time is required for the fluid to reach a given wetted length in a saturated condition.

The successful correlation between the empirical and numerical data in these limiting cases underscores the reliability of the numerical model. It also highlights the importance of considering saturation levels in the design and application of µPADs, as different saturation levels can significantly impact the fluid flow behavior within the paper substrate.

## 4. Discussion

This study has made significant contributions to the understanding of fluid dynamics in paper through both empirical investigations and numerical modeling. The findings presented here provide a comprehensive insight into the complex behavior of fluid flow in paper-based assays, addressing several key aspects crucial for their application and design.

### 4.1. Insights into Fluid Behavior across Saturation Levels in µPADs

One of the most critical insights from our study is the understanding of fluid behavior at different saturation levels, as presented in Figure 6. Our empirical results highlighted the importance of controlling saturation levels to ensure accurate and reliable fluid flow in µPADs. This understanding is especially crucial in applications requiring precise fluid manipulation, such as health diagnostics and environmental monitoring. The inability to accurately control fluid flow at higher saturation levels, as observed in our experiments, underscores the need for careful design and operational considerations in microfluidic applications.

### 4.2. Predictability and Applicability of the Numerical Model

The numerical model developed in this study has demonstrated its capability to predict fluid behavior across a range of saturation levels, as presented in Figure 7, particularly where empirical data were limited due to experimental constraints. The successful prediction of fluid flow, especially at higher saturation levels, validates the model and highlights its potential as a powerful tool for designing and optimizing µPADs. This is particularly relevant for situations where experimental approaches are not feasible or practical.

### 4.3. Impact of Pore Size and Fiber Orientation on Fluid Dynamics

The parametric study showing the impact of pore size in Figure 8 and fiber orientation in Figure 9 on fluid flow within filter paper has provided valuable insights into how these factors influence fluid dynamics. The ability of our model to predict changes in fluid flow with variations in pore size and fiber orientation demonstrates its versatility and applicability. This aspect is vital for the design of paper-based assays tailored for specific applications, where the substrate’s microstructural properties play a significant role in its functionality.

### 4.4. Validating the Numerical Model through Empirical Data

The findings presented in Figure 10 are significant, as they not only validate the numerical model but also provide insight into the dynamics of fluid flow in paper. The increasing slope with higher saturation levels reflects the influence of saturation on the fluid flow within the paper substrate. This relationship is crucial for applications where the precise control of fluids is necessary, such as in diagnostic tests or environmental monitoring using paper-based devices. The fidelity of our numerical model is further substantiated by the relative error calculations for various saturation levels, specifically a=0.00, 0.36, and 0.42. The mean relative errors were found to be approximately 5.89%, 6.46%, and 7.45%, respectively. Notably, these percentages are within the acceptable range of accuracy, often benchmarked at below 10%, affirming the robustness and reliability of our model. The agreement between the empirical observations and numerical predictions fortifies the model’s credibility and further accentuates the critical role of accurately accounting for saturation levels in the design and application of µPADs. Such considerations are essential since saturation levels have a profound impact on the internal fluid dynamics of paper substrates, which, in turn, affect the overall performance of µPADs.

### 4.5. Advancing µPAD Design: Implications from Fluid Dynamics Studies

The comprehensive understanding of fluid flow in µPADs gained through this study has several implications for their design and functionality. For instance, the realization that fluid flow is significantly affected by saturation levels and substrate properties such as pore size and fiber orientation can guide the development of more efficient and versatile assays. This knowledge can be used to enhance the diagnostic capabilities of paper-based devices, making them more adaptable to a range of applications. The integration of simulation tools has underscored their pivotal role in streamlining the design of POCT devices, enabling the nuanced control of fluid behavior essential for sophisticated diagnostic applications. Yet, the journey from simulation to practical application is marked by the imperative to rigorously validate theoretical models with real-world experiments, ensuring that our advancements in µPAD technology are both innovative and grounded in empirical evidence.

Future research should focus on further refining the numerical model to incorporate additional variables and conditions that may affect fluid flow in different paper substrates. Exploring the interplay between different substrate materials, fluid properties, and external conditions will deepen our understanding and enable the development of more sophisticated and application-specific designs. Moreover, incorporating multi-physical field simulations into our model will validate its universality and applicability, allowing for a comprehensive analysis of complex fluid interactions. Extending the model to simulate multi-fluid interactions and reactions within µPADs could open new avenues in multiplexed assays and complex analytical applications. In summary, this study bridges the gap between theoretical predictions and practical applications of fluid dynamics in paper-based assays. By providing a more robust and comprehensive model, we contribute to the fundamental understanding of fluid behavior in porous media and pave the way for innovative developments in µPAD technology.

## 5. Conclusions

This study represents a significant advancement in our understanding of fluid dynamics within paper-based analytical devices, which was achieved through a blend of empirical investigations and a comprehensive numerical model. We have elucidated the critical influence of parameters such as saturation levels, pore size, and fiber orientation on fluid flow in paper. Our empirical findings highlighted a distinctive relationship between saturation levels and wetted length, particularly at full saturation, where fluids lead to a marked slowdown in movement within filter paper.

The developed numerical model, incorporating the HSMAC method and the extended HORNET scheme, has demonstrated exceptional proficiency in predicting fluid flow behaviors that were challenging to obtain experimentally, especially at higher saturation levels. The model’s accuracy was further evidenced in parametric studies, which illustrated the impact of different pore sizes and fiber orientations on fluid dynamics, showcasing the model’s versatility. Quantitatively, the model effectively mirrored the Lucas–Washburn relationship for dry paper and revealed an increasing time requirement for the fluid to attain specific wetted lengths with rising saturation levels. This was quantified by the increasing slope in the linear relationship observed in the plots of the square of wetted length against time. Notably, the model accurately predicted a faster fluid flow in Whatman Grade 4 filter paper compared with Grade 41 due to its larger pore size. Additionally, it forecasted an increased flow rate in the machine direction fiber orientation of Whatman Grade 41. The insights from this study have critical implications for the design and practical application of µPADs. Understanding fluid behavior at different saturation levels, along with the substrate’s microstructural properties, can greatly enhance the efficiency and versatility of paper-based assays across various applications, including health diagnostics and environmental monitoring. Future work should aim to extend the model’s capabilities, incorporating additional variables, such as multi-fluid interactions, to further broaden the scope and utility of µPADs.

In conclusion, this research not only fills a significant gap in our knowledge of fluid flow in paper but also provides a robust framework for their future development and optimization. The combination of detailed empirical data with advanced numerical modeling marks a considerable stride in paper-based microfluidics, paving the way for novel applications and technologies in this field.

## Figures and Tables

**Table 1:** Parameters Used in Fluid Flow Analysis of Saturated Paper Substrates.

Variable	Value	Description/Reference
$\psi^{\prime }$	2.8	Diffusion Coefficient of W41 ^1^ Filter Paper [20]
$\phi_{dry}$	0.75	Porosity of Dry W41 ^1^ Filter Paper
C/D	9	Parameter for LW Flow in W41 ^1^ Filter Paper [21]
v	$1\times 10^{-6}$ $m^{2}/s$	Kinematic Viscosity of Water ^2^
ρ	$1000\;kg/m^{3}$	Density of Water ^2^

^1^ Whatman Grade 41 Filter Paper. ^2^ Water Servers as Both Primary (Saturating) and Secondary (Loading) Fluid.

**Table 2:** Empirical Fluid Flow Rate in Whatman Grade 41 and Whatman Grade 4 Filter Papers [18].

Wetted Length (*l*) [mm]	0.0	4.0	8.0	12.0	16.0	20.0
Time for Whatman 41 (*t*) [s]	0.0	1.1	4.8	11.9	22.9	39.0
Time for Whatman 4 (*t*) [s]	0.0	1.2	4.4	8.9	15.7	23.0

**Table 3:** Empirical Fluid Flow Rate in Whatman Grade 41 Filter Paper for Fiber Orientation in Cross-Machine Direction (CMD) and Machine Direction (MD) [18].

Wetted Length (*l*) [mm]	0.0	4.0	8.0	12.0	16.0	20.0
Time for CMD (*t*) [s]	0.0	1.1	4.8	11.9	22.9	39.0
Time for MD (*t*) [s]	0.0	0.8	4.0	9.9	19.3	32.4

## Data Availability

Data are contained within the article. Additional data not presented in this article are available on request from the corresponding author.
